# Supplementary material for: Fluctuation spectra of large random dynamical systems reveal hidden structure in ecological networks
Source: Nat Commun. 2021 Jun 15;12:3625. doi: 10.1038/s41467-021-23757-x (PMC8206210; doi:10.1038/s41467-021-23757-x)
Supplement: Supplementary file 1 — Reporting Summary [file 41467_2021_23757_MOESM1_ESM.pdf]

nature research

Corresponding author(s): Tim Rogers

Last updated by author(s): 11 May 2021

Reporting Summary

Nature Research wishes to improve the reproducibility of the work that we publish. This form provides structure for consistency and transparency in reporting. For further information on Nature Research policies, see our Editorial Policies and the Editorial Policy Checklist.

Statistics

For all statistical analyses, confirm that the following items are present in the figure legend, table legend, main text, or Methods section.

n/a	Confirmed	
☐	☒	The exact sample size (n) for each experimental group/condition, given as a discrete number and unit of measurement
☒	☐	A statement on whether measurements were taken from distinct samples or whether the same sample was measured repeatedly
☒	☐	The statistical test(s) used AND whether they are one- or two-sided<br>*Only common tests should be described solely by name; describe more complex techniques in the Methods section.*
☒	☐	A description of all covariates tested
☒	☐	A description of any assumptions or corrections, such as tests of normality and adjustment for multiple comparisons
☐	☒	A full description of the statistical parameters including central tendency (e.g. means) or other basic estimates (e.g. regression coefficient) AND variation (e.g. standard deviation) or associated estimates of uncertainty (e.g. confidence intervals)
☒	☐	For null hypothesis testing, the test statistic (e.g. F, t, r) with confidence intervals, effect sizes, degrees of freedom and P value noted<br>*Give P values as exact values whenever suitable.*
☒	☐	For Bayesian analysis, information on the choice of priors and Markov chain Monte Carlo settings
☒	☐	For hierarchical and complex designs, identification of the appropriate level for tests and full reporting of outcomes
☒	☐	Estimates of effect sizes (e.g. Cohen's d, Pearson's r), indicating how they were calculated

Our web collection on statistics for biologists contains articles on many of the points above.

Software and code

Policy information about availability of computer code

Data collection	Simulation data are produced with Matlab (2019b) and Python code provided at: http://doi.org/10.5281/zenodo.4720998
Data analysis	Data analysis code (Matlab 2021b) for Fig 5 is provided at: http://doi.org/10.5281/zenodo.4720998

For manuscripts utilizing custom algorithms or software that are central to the research but not yet described in published literature, software must be made available to editors and reviewers. We strongly encourage code deposition in a community repository (e.g. GitHub). See the Nature Research guidelines for submitting code & software for further information.

Data

Policy information about availability of data

All manuscripts must include a data availability statement. This statement should provide the following information, where applicable:

- Accession codes, unique identifiers, or web links for publicly available datasets
- A list of figures that have associated raw data
- A description of any restrictions on data availability

Plankton abundance data used in Fig 5 are taken from reference [34] available at: nature.com/articles/s41467-017-02571-4\#Sec24.
All simulation data can be reproduced using the code available at: http://doi.org/10.5281/zenodo.4720998

Field-specific reporting

Ecological, evolutionary & environmental sciences study design

All studies must disclose on these points even when the disclosure is negative.

Study description	A theoretical study of fluctuations in large random ecosystem models.
Research sample	Random Lotka-Volterra ecosystems, generated using Matlab 2019b.
Sampling strategy	Theory figures show single samples and are for illustration purposes only - our theoretical results are exact in the limit of large system size. Figure 5 shows results from a real data set described in reference [34].
Data collection	Simulated time-series generated using Matlab 2019b. Data collection details for Fig 5 are in ref [34].
Timing and spatial scale	Theory results: all units are arbitrary. Data used in Fig 5 (from ref [34]) were collected over 88 consecutive days.
Data exclusions	No exclusions from simulations. Species with instances of zero abundance were removed from data set for Fig 5.
Reproducibility	Code to reproduce all figures is available at: http://doi.org/10.5281/zenodo.4720998. Theory work can be reproduced directly from the manuscript.
Randomization	Not applicable.
Blinding	Not applicable.

Did the study involve field work? ☐ Yes ☒ No

Reporting for specific materials, systems and methods

We require information from authors about some types of materials, experimental systems and methods used in many studies. Here, indicate whether each material, system or method listed is relevant to your study. If you are not sure if a list item applies to your research, read the appropriate section before selecting a response.

Materials & experimental systems

n/a	Involved in the study
☒	☐ Antibodies
☒	☐ Eukaryotic cell lines
☒	☐ Palaeontology and archaeology
☒	☐ Animals and other organisms
☒	☐ Human research participants
☒	☐ Clinical data
☒	☐ Dual use research of concern

Methods

n/a	Involved in the study
☒	☐ ChIP-seq
☒	☐ Flow cytometry
☒	☐ MRI-based neuroimaging
